# Supplementary material for: The interactome of intact mitochondria by cross-linking mass spectrometry provides evidence for coexisting respiratory supercomplexes
Source: Mol Cell Proteomics. 2017 Dec 8;17(2):216–32. doi: 10.1074/mcp.RA117.000470 (PMC5795388; doi:10.1074/mcp.RA117.000470)
Supplement: Supplemental Data [file supp_RA117.000470_133922_0_supp_23978_fzffwf.zip › spectra_annotation/mito_spectra_annotation/435-1-8-1-15-1.pdf]

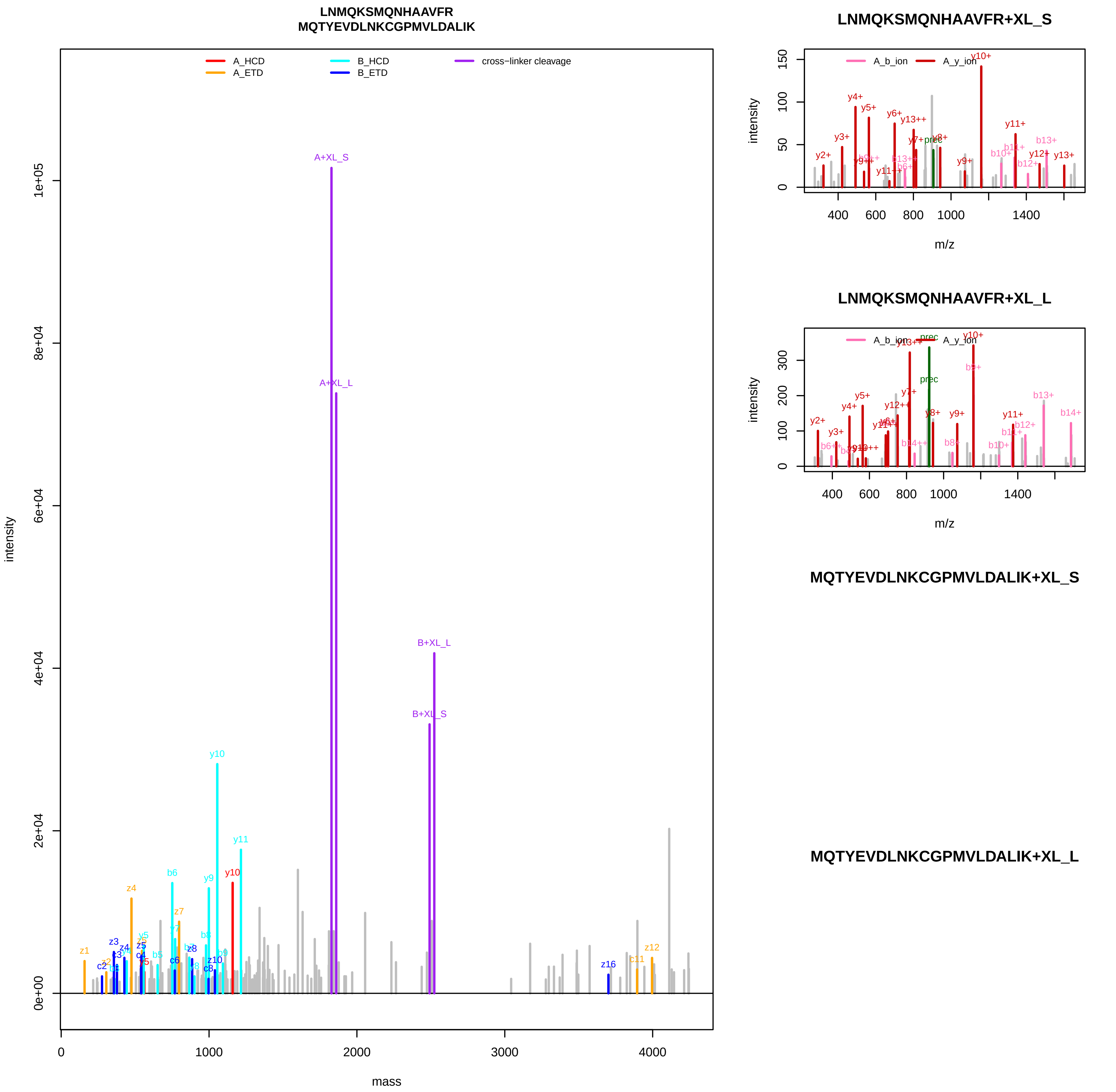
LNMQKSMQNHAAVFR
MQTYEVDLNKCGPMVLDALIK
A_HCD
A_ETD
B_HCD
B_ETD
cross−linker cleavage
A+XL_S
A+XL_L
B+XL_L
B+XL_S
y10
y11
y9
b6
y10
z4
z7
z12
z16
z11
intensity
mass
0e+00
2e+04
4e+04
6e+04
8e+04
1e+05
0
1000
2000
3000
4000
LNMQKSMQNHAAVFR+XL_S
A_b_ion
A_y_ion
intensity
m/z
LNMQKSMQNHAAVFR+XL_L
A_b_ion
A_y_ion
intensity
m/z
MQTYEVDLNKCGPMVLDALIK+XL_S
MQTYEVDLNKCGPMVLDALIK+XL_L
